# Challenges in the diagnosis and management of anti-phospholipid syndrome: a case from Cameroon

**DOI:** 10.1186/s13104-017-2689-3

**Published:** 2017-08-08

**Authors:** Ahmadou M. Jingi, Liliane Mfeukeu-Kuate, Aurel T. Tankeu, Narcisse Assene Ateba, Edvine Wawo Yonta, Jean Jacques Noubiap

**Affiliations:** 1 Department of Internal Medicine and Specialties, Faculty of Medicine and Biomedical Sciences, University of Yaoundé I, Yaoundé, Cameroon; 2 Division of Cardiology, Yaoundé Central Hospital, Yaoundé, Cameroon; 3 Department of Medicine, University of Cape Town and Groote Schuur Hospital, 7925 Observatory, Cape Town, South Africa

**Keywords:** Anti-phospholipid syndrome (APLS), Cameroon, Sub-Saharan Africa (SSA), Thrombosis

## Abstract

**Background:**

Anti-phospholipid syndrome (APLS) is a condition characterized by the presence of raised plasma levels of anti-phospholipid antibodies associated with thrombo-embolic disease and/or poor obstetrical outcomes in women. The epidemiology of APLS is unknown in most sub-Saharan African countries due to limited access to diagnosis tools.

**Case summary:**

We report the case of APLS in a 29-year-old obese woman that was preceded by pre-eclampsia and fetal death. The diagnosis of APLS was made during a thrombo-embolic episode 4 years after the poor obstetrical outcome. Her management was challenging, as she had three thrombo-embolic events within 18-months despite treatment with anti-coagulant (acenocoumarol).

**Conclusion:**

This case highlights the need for screening for APLS after an episode of hypertensive disease in pregnancy or fetal death, and the challenges faced with the treatment, such as resistance to antivitamin K anti-coagulants and the desire for maternity.

## Background

Anti-phospholipid syndrome (APLS) is a condition characterized by the presence of raised plasma levels of anti-phospholipid antibodies associated with thrombo-embolic disease and/or poor obstetrical outcomes in women [1]. APLS can occur as an isolated form, termed “primary APLS” or Hughes syndrome [1], or in association with other auto-immune conditions. The epidemiology of APLS in our low-income setting is unknown, probably due to under-diagnosis and under-reporting. Indeed, the diagnostic tests for APLS are unavailable in most health facilities and the treatment options are limited [2]. We present here the second reported case of APLS in Cameroon, and highlight challenges in diagnosis and management.

## Case presentation

Miss E.S., a 29-year-old lady, presented to us on December 29, 2016, with one day history of insidious onset, painful and swollen left lower limb in the absence of fever, interfering with her normal daily activities. Her past medical history was remarkable for severe pre-eclampsia with provoked fetal expulsion at 24 weeks of amenorrhea on December 24, 2011. No in-depth diagnostic work-up was carried out after this event. In March 18, 2015, she had an inaugural deep venous thrombosis (DVT) involving the left calf, complicated by bilateral proximal pulmonary embolism as shown by doppler ultrasound of the leg veins, and computed tomography pulmonary angiography. She was treated with anti-vitamin K (acenocoumarol), but had two other thrombo-embolic events within 2 months despite antivitamin K treatment (total of three episodes within 18 months).

**Fig. 1:**
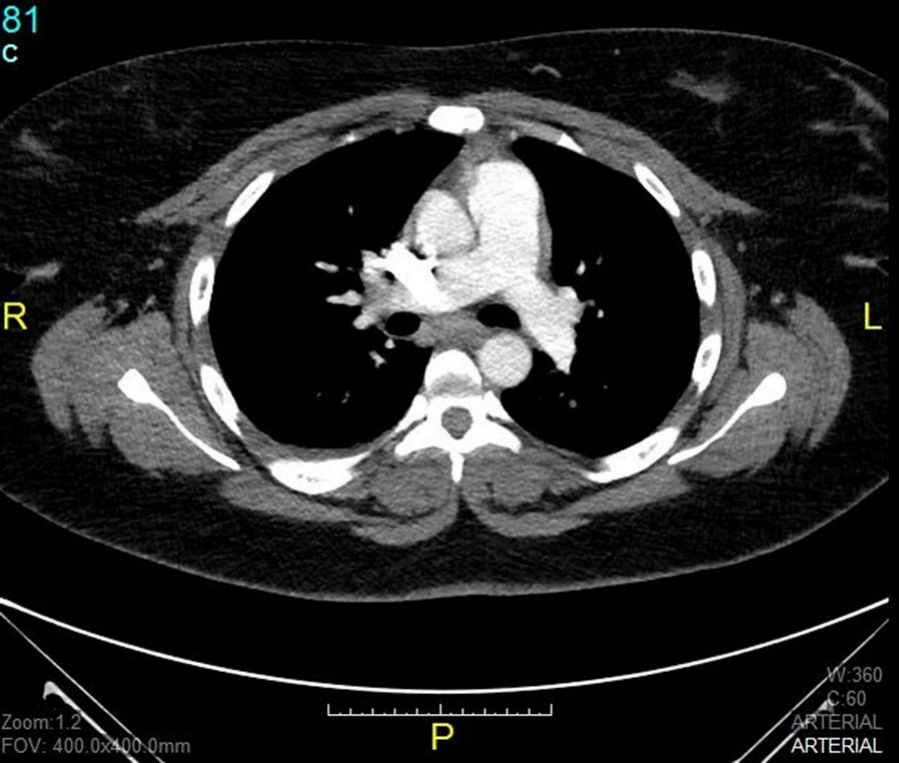
CT scan

The recurrence of thrombo-embolic events prompted her referral to a specialized centre where she complained of grade 3 New York Heart Association (NYHA) dyspnea, orthopnea and pleuritic chest pain. On clinical examination she had a blood pressure of 130/90 mmHg, regular pulse at 100 bpm, saturation of 95% on ambient air. Her body mass index (BMI) was 44.5 kg/m^2^. Chest auscultation revealed inspiratory crackles at the right lung base. She had post-phlebotic left lower leg, and the rest of examination was unremarkable. Pulmonary function tests showed a restrictive pattern with a best forced vital capacity (FVC) of 1.4 l (40% of predicted), a total lung capacity of 60%, with a predicted residual volume of 100%. The diffusing capacity of the lungs for carbon monoxyde (DLCO) was reduced to 44%. Electrocardiogram showed regular sinus tachycardia at 100 bpm. A chest X-ray showed normal cardiothoracic index, bilateral basal atelectasis, right lower zone consolidation, and small bilateral pleural effusions. Cardiac ultrasound showed normal heart chambers, with normal systolic function (ejection fraction of 72%). The pulmonary systolic pressure was estimated at 28 mmHg. A computed tomography of the pulmonary arteries showed a large pulmonary embolus in the right middle and lower lobes, with post-embolic consolidation and atelectasis (Fig. 1). Concomitant doppler ultrasound of the lower limbs was negative for DVT. Blood tests showed a mild microcytic anemia with hemoglobin level of 12.5 g/dl. The international normalized ratio (INR) was 1.5, with normal thyroid, liver and kidney functions. Human immunodeficiency virus (HIV) and hepatitis C serology was negative, and viral hepatitis B test showed immunity to a previous natural infection. Her anti-phospholipid antibodies were positive on three occasions (anti-cardiolipin IgA at 24 APL U/ml and anti-β2GP 1 IgA at 43 AEU/ml), and a genetic screen was negative for Factor V Leiden and Prothrombin gene mutation. Antinuclear factor was also negative. However, a previous test (June 14, 2015) prior to visiting this center was positive for anti-DNA antibody at 54 IU/ml, with anti-nuclear factor at 80 IU. Protein C and S titers were low (79% and 51% respectively). Blood sample was however drawn shortly after commencing acenocoumarol. She was then treated with high dose subcutaneous enoxaparin 100 mg twice daily, and then switched to rivaroxaban after 10 days.

Evolution on treatment was marked by improvement in the symptoms (functional class 2, ambient air saturation of 96%), respiratory function tests (FVC of 2.9 l [84% of predicted]), and chest X-ray (resolution of atelectasis and effusion). Otherwise, Miss E.S. was sedentary (Bank agent), non-smoker, and was not on oral contraceptive pills. She was on acenocoumarol 4 mg daily at consultation, which she has been taking for the past three months (October 2016). She reverted to acenocoumarol after the 6 month course of Rivaroxaban, pending her clinical appointment. INR was not regularly checked and she was not on any diet. A distant relative (father’s cousin) had DVT at 56 years of age with no in-depth investigation for thrombophilia.

On December 29, 2016, when we saw her with the complaint of one day history of insidious onset painful and swollen left lower limb, she also reported having episodic chest pain around the right clavicle and pelvic pain during defecation, evolving since 1 week. On examination she had morbid obesity (BMI of 44.5 kg/m^2^, and abdominal circumference of 119 cm) and acanthosis nigricans suggesting insulin resistance. She has the following vital parameters: BP of 128/90 mmHg, heart rate of 107 bpm, respiratory rate of 28 cpm, and saturation of 100% in ambient air. Her left lower limb was markedly swollen (thigh difference of 14 cm), with patchy areas of petechiae.

**Fig. 2:**
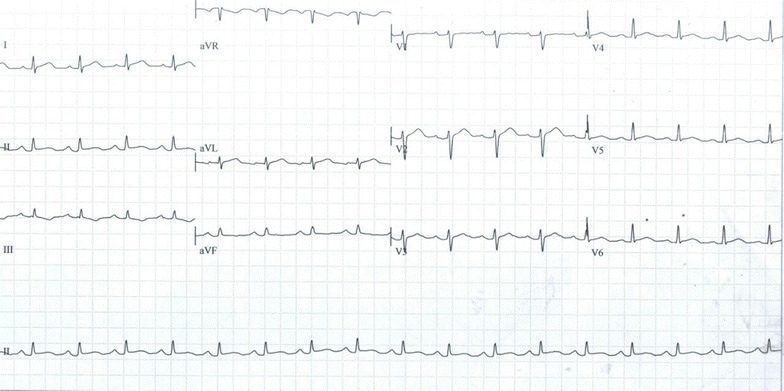
Electrocardiogram

Recurrence of thromboembolic disease was suspected, which was confirmed by doppler ultrasound of the left lower limb. Cardiac ultrasound was unremarkable, and electrocardiogram (ECG) showed regular sinus tachycardia at 102 bpm, with an S1Q3T3 pattern (Fig. 2). Full blood count showed persistent mild microcytic and hypochromic anemia (Hb of 10.1 g/dl), with thrombocytopenia (platelets of 123,000/mm^3^). The INR was 1.45 on 4 mg of acenocoumarol. Work up for metabolic syndrome showed an HbA1c of 6%. Lipid profile was remarkable for mildly elevated low density lipoprotein cholesterol (LDLc) at 1.31 g/l, and serum urate was 51.4 mg/l. She was treated with high dose of subcutaneous low molecular weight heparin (enoxaparin).

## Discussion

We present the case of APLS in a 29-year-old obese woman who initially presented with severe pre-eclampsia four years prior to an inaugural thrombo-embolic event that led to the diagnosis. She had recurrent episodes of thrombo-embolic events despite taking anti-vitamin K.

This is the second documented case of APLS in our country after that reported by Luma et al. in a 43-year-old man [2]. This suggests under diagnosis and under reporting of APLS, in the context of limited access to diagnostic tests. Thus, the exact burden of APLS remains unknown in sub-Saharan African countries.

The management of APLS is challenging in our context of limited resources where there is no universal health coverage [3, 4]. Once diagnosed, APLS requires chronic and sometimes expensive treatment, regular laboratory checks, and dietary modifications. Unfortunately, this usually results in non-adherence to treatment, medical visits, and laboratory checks due to financial limitations. This is especially frequent in patients treated with anti-vitamin K anti-coagulants. In our case, the patient, despite being on medical aid., missed many medical visits and laboratory tests while on acenocoumarol. This non-compliance was even worse in the case reported by Luma et al. since their patient did not probably benefit from any form of medical aid [2]. Even in treated patients, especially with the more available and affordable acenocoumarol, the risk of recurrent thrombo-embolic events remains high as shown in our case (treated with acenocoumarol) and that reported by Luma et al. (treated with aspirin). This could be due to a high rate of resistance to the anti-vitamin K anticoagulants or aspirin in our patients presenting an APLS [5, 6]. However, the exact burden of anticoagulant or aspirin resistance in our setting is unknown. Importantly, patients with APLS should also be screened for other comorbid conditions that can lead to hypercoagulability states, or worsen outcome. Our patient was suspected of having metabolic syndrome. She had morbid obesity, with skin signs of insulin resistance (acanthosis nigricans) [7], pre-diabetes, and mildly elevated serum levels of LDLc. The chronic microcytic and hypochromic anemia could be due to APLS, occult blood lose as a result of anticoagulation, or chronic inflammation. Management of her anemia remained challenging.

## Conclusion

This case highlights the need for screening for APLS after an episode of hypertensive disease in pregnancy or fetal death, and the challenges faced with the treatment, such as resistance to antivitamin K anti-coagulants and the desire for maternity. Combination treatment with anti-coagulants, anti-platelets, and biotherapy could be used in patients with recurrent thrombo-embolic disease. Local resources for the diagnosis and management of APLS should be scaled up in health facilities in sub-Saharan African countries.
